# Supplementary material for: A bio-mimetic miniature drone for real-time audio based short-range tracking
Source: PLoS Comput Biol. 2022 Mar 8;18(3):e1009936. doi: 10.1371/journal.pcbi.1009936 (PMC8932603; doi:10.1371/journal.pcbi.1009936)
Supplement: Attachment — Submitted filename: Reviewer_answer_Jan_27_2022.docx [file pcbi.1009936.s003.docx]

*Reviewer #1: In the manuscript titled “A bio-mimetic miniature drone for real-time audio based tracking” the authors report a study on a performance of a small autonomous flying robot that uses two microphones, as well as a custom-developed microcontroller, to locate the orientation of a sound emitter. By orienting towards the sound source, the drone could follow a single speaker – given that the motion speed of the source is small enough so that the drone does not lose it, and its velocity does not change very abruptly. The authors discuss the performance and the limitation of their approach to some extent. The manuscript is generally well written, it is clear and easy to follow.
This is a very exciting line of research, in my opinion, but I have several crucial concerns, which honestly makes it hard for me to give a clear recommendation.*

We thank the reviewer for the helpful comments

*Major concerns:
I am not convinced that the topic of the manuscript fits well in the field of Computational Biology. Although it’s connection to biology in its aims are obvious, the manuscript does not discuss any biological implications in detail. It is also not clear whether the actual implementation could or could not be used to study animals. The manuscript may better suit in a more technological journal?*

We agree that the paper would be of much interest to technological journals as well, but we are convinced that it is suitable to PLoS Computational Biology too. The approach taken in the study and the entire discussion takes a biological point of view, and the system itself is bio-mimetic - an engineering approach would have used more than two microphones and would have spaced them farther apart to ease localization while we intentionally did not do this in order to be similar to the biological situation. Moreover, the approach of studying nature through physical robotics (rather than computer simulations) is on the rise and has several clear advantages as we stress in the paper. In the revised manuscript, we added more discussion of the biological relevance in several places along the manuscript.

*The title is not specific enough. It does not specify that the method could only work for locating a single sound source and within a very short range. I am sceptical about how the current implementation could overcome these limitations, so at least the title should clearly state them somehow.*

We added short-range to the title. The approach could actually be elaborated to dealing with more than one source, for instance, by adding an algorithm that localizes multiple sounds and chooses among them (or takes some weighted average of their direction). This is actually what we are working on these days. In fact, in a previous robotic work, we have shown how multiple sound-sources (in that case echoes) can be localized within a single sound-recording. We added this to the discussion (lines 143-152).

*The method they use and report in this manuscript works for sound localisation only in 2D. Although the authors point this out in their discussion (2nd paragraph), but I think it is a major limitation that should be made clear from the beginning. Generalizing the system to work in 3D (using only 2 microphones) would be a hard challenge.*

The author is right that the system currently only works in 2D and we now added this information early-on in the manuscript (lines 24-26). However, it should not be difficult, in theory, to generalize the approach to 3D by adding pinnae on-top of the microphones. As well documented in mammals, such pinnae provide elevation-specific filtering which complete the time of arrival differences and provide 3D information. In fact, we are currently implementing such an approach in a terrestrial robot. This addition could be also implemented to the drone. We now explain this in the discussion (lines 138-142).

*Minor comments:
Fig. 1c. The box for the keys is misaligned. Using “drone” and “source” here is a much better choice than “Crazyflie” and “Speaker” as on Fig. 2. This inconsistency should be resolved.*

Corrected

*Page 8, last line: N should be given for the Pearson correlation.*

Corrected (line 108).

*Page 9, Fig. 2. Legend, last line: “Of the 20 trials that were recorded the seven that had most consistent video tracking were analyzed.” This sounds worrying, as it is not clear how the presented data was selected.*

Indeed, we used a bad description. Of the 20 trials we ran only eight contained usable data. In the other trials there was no data for various technical reasons: either the tracking system did not start, the drone’s battery finished or the drone exited the FOV covered by the system, etc. We now clarify this in the revised methods. Note that the visual system’s tracking errors have nothing to do with the sound tracking performance of the drone Lines (207-210).

*At page 11, the Authors state about the tracking: “Motion was tracked at 200 fps and with a spatial resolution of less than 1 mm.” So either there they should report any other source of tracking error or proportion of missing track points, or they should report results calculated for an entire recorded data set.*

We provided a reference that provides all of the necessary information to support this statement. The reference was already present in the previous version, but we now clarify this in the revised manuscript (lines 205-206).

*Some of the abbreviations are not introduced.*

Corrected

*In the 3rd paragraph of the discussion, the Authors refer to an unpublished work (Ref. 24. Elyakim, I., Kosa, G. & Yovel, Y. An autonomous navigating and mapping acoustic robat. Under review. ). Or is this meant to be a reference to Elyakim, I., Cohen, Z., Kosa, G. & Yovel, Y. A fully autonomous terrestrial bat-like acoustic robot, PLOS Computational Biology, 14.9 (2018)?*

This should be the same (published) paper. We corrected this.

*Page 11, equations: The two equations should match in their appearance. If tau_difference is defined in the first equation, I see no reason why this is not used in the second equation.*

Corrected (lines 181 and 189).

*I think the figure titles could be improved by changing them to summarise what is illustrated on the figure, and not just state what the drone did.*

We improved the axes titles and hopefully they are clear now.

*Typos:
Fig. 2. Legend, line 2. “in2-Dimentional”*

All typos were corrected.

*Summary*
*As a consequence of all the above, I can’t recommend publication in PLOS Computational Biology.*

*Reviewer #2: This paper describes a biomimetic robot inspired by bats that uses two microphones and a time difference of arrival approach to track and turn toward a speaker producing high-frequency pings. Two experiments are performed to demonstrate the capabilities of the robot - one where the robot remains stationary and turns toward the speaker, and one where it moves with a constant velocity and pursues the speaker. The article is clearly written and the results demonstrates proof-of-principle that the system can perform this tracking task reasonably well. As I am not an expert in robotics or sound localisation, I cannot assess the novelty of the approach, however I found the paper interesting and the results seem sound. I have a few suggestions for improvement.*

We thank the reviewer for the helpful comments

*1. The authors provide a link to a Github repository containing the source code for the project. I am glad to the code is available, however there is very little documentation, which would make it difficult for someone to reproduce. I think this issue is especially relevant as the paper is really about demonstrating a method as a proof of principle, thus a large part of the value would be in allowing other researchers to use and expand on the system. I would therefore suggest better documentation to allow potential replication of the setup.*

We now heavily document the code. If the reviewer has further suggestions, we would be happy to apply them.

*2. It is mentioned in the manuscript that a tracking system using 24 cameras was used to track the drone and playback speaker. Is this system described elsewhere? If so, it would be good to provide a reference, and if not, I think more detail should be included on the tracking system.*

The system was thoroughly tested in the past. This information can be found in supplementary figure 1 in Eitan et al. 2019. We now refer the readers to this information more specifically (lines 203-206).

*3. The tests have been carried out by a human manually moving a speaker around. This probably introduces some bias into the measurements, as the human (who probably wants the robot to succeed) may subtly respond to the robot by slightly modifying their behavior, e.g. to slightly wait for it if it is behind. The ideal case for testing would have been to have the speaker movement automated, however I recognise this might have been more trouble than it is worth. Overall, I don’t see this as a huge issue as the paper mainly serves as a proof of concept, but it might be worth just acknowledging this as a potential limitation.*

We refer to this point in the manuscript. Because the drone is currently going through several improvements, we could not run the suggested control, but we agree that this is a good point and now refer to it in the discussion (lines 148-152).

*Line edits:

As a general comment, it would be nice to provide line numbers to simplify review. I have done my best here to describe where these edits are located.*

We apologize and added line numbers.

*Pg 1 paragraph 3: “theoretic” should probably be “theoretical”*

Corrected (line 51).

*Pg 2 Results: “hovering at place” —> “hovering in place”*

Corrected (line 83).

*Pg 6 “the system allowed to record” —> “the system allowed recording of” or “the system allowed us to record”*

Corrected (line 176).

*Figure 2: I suggest labelling the legend as “drone” rather than “crazyflie” for clarity*

Corrected
